# Fluid Biomarkers in Demyelinating Spectrum Disorders: Past, Present, and Prospects

**DOI:** 10.3390/ijms26094455

**Published:** 2025-05-07

**Authors:** Anca-Maria Florea, Monica Neațu, Dimela-Gabriela Luca, Eugenia Irene Davidescu, Bogdan-Ovidiu Popescu

**Affiliations:** 1 Department of Clinical Neurosciences, “Carol Davila” University of Medicine and Pharmacy, 050474 Bucharest, Romania; anca-maria.florea@rez.umfcd.ro (A.-M.F.); monica.neatu@rez.umfcd.ro (M.N.); bogdan.popescu@umfcd.ro (B.-O.P.); 2 Department of Neurology, Colentina Clinical Hospital, 020125 Bucharest, Romania; dimela_luca@yahoo.com; 3 Department of Cell Biology, Neurosciences and Experimental Myology, “Victor Babes” National Institute of Pathology, 050096 Bucharest, Romania

**Keywords:** multiple sclerosis, neuromyelitis optica spectrum disorder, MOGAD, biomarker, disease-modifying therapy

## Abstract

The diagnostic algorithm for the demyelinating disorders of the central nervous system remains a work in progress, with the search for the ideal biomarkers ongoing. The so-called “ideal” biomarker should ensure the accurate differentiation between the most common demyelinating pathologies of the CNS and between the subtypes of the same pathology (for example, the conversion from relapsing–remitting multiple sclerosis to the secondary progressive phenotype). Advances in technology facilitated this research and in the following sections we will comprehensively review most of these, outlining the past, present, and prospects and the impact they had on both diagnosis and therapeutic approach.

## 1. Introduction

Inflammatory demyelinating diseases of the central nervous system (CNS) have captivated interest since Charcot first described multiple sclerosis in 1868 [1,2], owing to the intricate interplay between their underlying pathophysiology and clinical manifestations. To this day, these conditions remain among the leading causes of disability in Neurology. According to the published data, up to 2.8 million people worldwide live at the moment with pathologies that fall within the spectrum of demyelinating disorders [3]. Among these, the most common ones, which make the subject of the current paper, are multiple sclerosis (MS), neuromyelitis optica spectrum disorder (NMOSD), and myelin oligodendrocyte glycoprotein-associated disease (MOGAD). In addition to these, Balo’s concentric sclerosis, Schilder’s disease, and Marburg disease are worth mentioning, being recognized as variants of these diseases [4]. The correct diagnosis of these disorders is essential to establish realistic expectations about disease progression and to avoid inappropriate treatment.

The first study on biomarkers dates back to the 1950s, with the discovery of immunoglobulin IgG in the cerebrospinal fluid (CSF) of MS patients [5]. Ever since, scientists have continuously drawn attention to different fluid biomarkers to stratify diagnosis and management, focusing on targeted treatment approaches. Despite the CSF being considered the gold standard for detecting biomarkers, the use of plasma, saliva, and urine has recently gained interest, especially due to the advances in technologies such as genomics, proteomics, and transcriptomics [6].

## 2. Current Biomarkers of Demyelinating Diseases

In this concise review, we examine the biomarkers specific to CNS demyelinating disorders and trace their historical development. To compile the literature, we conducted searches of the following databases: PubMed, Scopus, and Google Scholar. We focused our research on articles published in English, mainly between 2018 and 2025. Key search terms included “biomarker”, “demyelinating disorders of the central nervous system”, “multiple sclerosis”, “NMOSD”, and “MOGAD”. We selected studies that addressed these terms collectively, as well as those detailing the specific functional role of each biomarker in the relevant pathologies. Publications not available in English were omitted from this review. The reference list of the included studies was subsequently examined to identify additional relevant publications.

Demyelinating diseases of the CNS can present with overlapping features, which can make diagnosis particularly challenging at the onset [7]. As previously noted, establishing an accurate diagnosis of autoimmune demyelinating disorders is essential for guiding therapeutic management and determining medium-to-long-term prognosis. Even though progress has been made in achieving diagnostic accuracy and concerning therapeutic approach, the search for the “ideal” biomarker is still a work in progress. The so-called “ideal” biomarker should guide clinicians in assessing disease activity, predicting the risk of relapses, and, based on these, choosing the appropriate disease-modifying therapy (DMT) [8].

This review focuses on the following central nervous system demyelinating disorders- summarized in Table 1 [4,9,10,11,12,13,14,15]:

*Multiple sclerosis (MS)* is a chronic pathology of the CNS, representing up to this day the leading cause of disability in young adults, with a mean age of onset of around 30 years [3]. The cause is unknown, but it appears that a synergy between environmental, genetic, and immune factors is implied in the pathology, which is characterized by demyelination, inflammation, neuronal loss, and gliosis [16].

The hallmark pathological features include focal lesions characterized by primary demyelination and astrocytic scarring, which emerge against a backdrop of chronic inflammation. Primary demyelination involves the destruction of myelin sheaths and their supporting oligodendrocytes, while axons remain partially intact. Despite this, significant axonal and neuronal damage occurs within both gray and white matter lesions. The severity of this injury serves as the strongest pathological indicator of irreversible neurological impairment [17].

Several variants of MS have been described, including clinically isolated syndrome (CIS), radiologically isolated syndrome (RIS), relapsing–remitting MS (RRMS), and progressive forms, that may arise either from onset (primary progressive MS—PPMS) or evolve from a remitting course over time (secondary progressive MS—SPMS) [2]. Distinguishing between PPMS and SPMS is essential for selecting the most effective treatment, predicting disease progression, and improving patient care [18]. Moreover, biomarkers for the early identification of the transition between clinically isolated syndrome and the relapsing–remitting form, as well as between the relapsing–remitting and chronic progressive forms, remain a subject of ongoing research [19].

*Neuromyelitis optica (NMO)* was first described in 1894 by Devic [20] and is a pathology distinct from MS regarding not only lesion morphology but also disease progression and therapeutic approach [2]. It is characterized by frequent relapses and permanent disability, which underscores the importance of early diagnosis. Demyelination predominantly involves the optic nerves and the spinal cord, which can make distinguishing it from MS particularly challenging in the early stages [21]. A major milestone in this field was the discovery of aquaporin-4 (AQP4) antibodies in 2004, which are highly specific for NMOSD patients. Additionally, demonstrating the association between myelin oligodendrocyte glycoprotein (MOG) IgG antibodies and NMOSD in AQP4-negative patients further enhanced the ability to identify affected individuals. However, accurately diagnosing double-seronegative patients remains an ongoing challenge [21,22].

*Myelin oligodendrocyte glycoprotein antibody-associated disease (MOGAD)* was first described in 2007 as a distinct entity. This demyelinating disorder may have a monophasic or relapsing course [23]. Individuals testing positive for MOG-IgG may present with clinical manifestations such as optic neuritis, transverse myelitis, acute disseminated encephalomyelitis (ADEM), or encephalitis with the involvement of the cerebral cortex, cerebellum, or brainstem [24]. Several investigations using cell-based assays with mammalian cells expressing the complete human MOG protein have emphasized the presence of MOG-IgG in patients with non-MS demyelinating disorders of the CNS. Moreover, these studies also revealed that 30–70% of patients with seronegative NMOSD tested positive for MOG-IgG [9].

**Figure 1:**
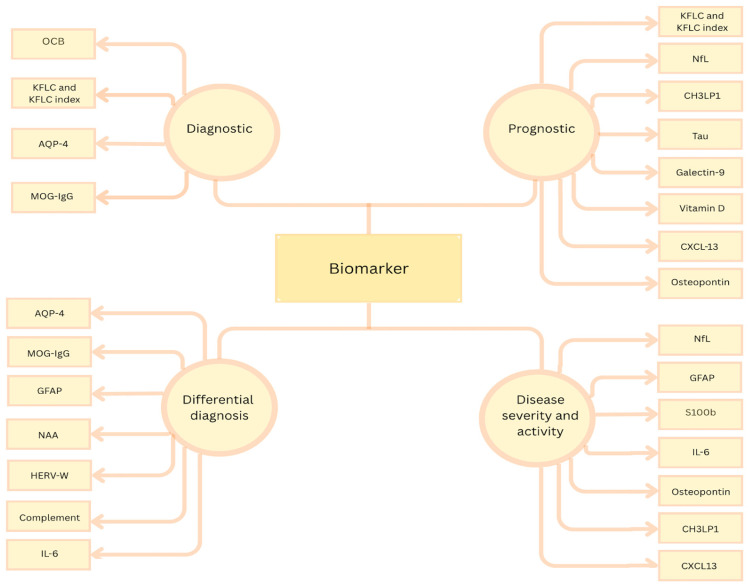
Biomarker classification according to their role; OCBs = oligoclonal bands; KFLC = kappa free light chain; AQP4 = aquaporin-4; MOG = myelin oligodendrocyte glycoprotein; GFAP = glial fibrillary acidic protein; NAA = N-acetyl aspartate; HERV = human endogenous retrovirus; IL-6 = interleukin-6; NfL = neurofilament light chain; CH3LP1 = chitinase-3-like protein 1; CXCL13 = (C-X-C) motif ligand 13; S100B = calcium binding protein.

To date, numerous biomarkers have been rigorously investigated for diagnostic or prognostic applications, with several serving multiple functions, as illustrated in the figure below (Figure 1) [25].

### 2.1. OCBs

Oligoclonal bands (OCBs) detected in cerebrospinal fluid serve as markers for the intrathecal synthesis of IgG and IgM immunoglobulins within the central nervous system. Over time, the methods for detecting these bands have advanced, with isoelectric focusing on agarose gel now regarded as the gold standard and immunodetection recognized as the reference method [26]. The research dates back to the 1950s–1970s, when the technology of CSF protein electrophoresis developed, defining positive OCB as the presence of more than two bands in the gamma globulin region [27].

Based on the CSF–serum correlation, five categories are described:

- Type 1: OCB-negative in both the CSF and serum;
- Type 2: OCB-positive in the CSF, and OCB-negative in the serum;
- Type 3: OCB-positive in both the CSF and serum, with additional bands in the CSF;
- Type 4: identical OCB in both the CSF and serum;
- Type 5: “Monoclonal bands” [27].

In multiple sclerosis, OCBs are present in more than 95% of patients, reflecting the humoral immunity involvement in the CNS through B cells [28]. The 2017 McDonald criteria emphasize the importance of the presence of bands by dispensing the need to fulfill the dissemination in the time criterion for diagnostic certainty. More than that, they are essential in the clinically isolated syndrome (CIS), where the presence of OCBs serves as an independent risk factor, nearly doubling the chance of progressing to clinically definite MS over an average follow-up period of 4 years [29].

The limitations become evident when making a differential diagnosis with other pathologies involving the CNS, such as Sjogren syndrome, neurosarcoidosis, systemic lupus erythematosus, paraneoplastic disorders, Behcet’s disease, neuroborreliosis, and so on [30]. It has also been stated that, in these cases, OCBs could be present for up to 2 years even with the pathology being quiescent [27].

Regarding the differential diagnosis with other demyelinating disorders, it is essential to outline the absence/low positivity rate of OCBs in AQP4-Ab-positive NMO, as studies proved no intrathecal synthesis of IgG [31]. Oligoclonal bands are found in only 10–25% of patients with NMO [27]. In the samples positive for OCBs, the amount of intrathecally produced IgG was below the second percentile and the OCBs could disappear in 6 months, which is uncharacteristic for multiple sclerosis [27,31].

### 2.2. Kappa and Lambda Free Light Chains and Kappa Free Light Chain Index

Kappa and lambda free light chains (KFLC, LFLC) represent biomarkers worth mentioning, and have been researched since the late 1970s. According to the latest data, assessing intrathecal KFLC synthesis has been included in the 2024 McDonald revised criteria: the diagnosis of MS can be made when the requirements for dissemination in space are fulfilled, with evidence of intrathecal IgG synthesis and either oligoclonal bands or kappa free light chains serving as an additional criterion [32].

As already known, immunoglobulin light chains are subunits of antibodies that are excessively produced during infectious or inflammatory processes. The kappa chain is particularly important because it is produced in the sheath of the CNS [3,4], and multiple studies have demonstrated that this isoform offers superior diagnostic utility over lambda free light chains in neuroinflammatory disorders [16]. In addition to the direct assessment of KFLC and LFLC in serum/CSF, another biomarker is represented by the KFLC index, whose value is obtained by the following formula: FLC index = QFLC/QALB (where QFLC = CSF FLC/serum FLC and QALB = CSF albumin/serum albumin) [3]. Since the index includes QALB, it is more precise in determining the intrathecally produced fractions and differentiating from the ones that diffused from the serum to the CSF [5].

According to the studies available up to this moment, KFLC quantification represents a rapid and economically efficient biomarker of intrathecal immunoglobulin synthesis (a comparable diagnostic accuracy to OCB but with substantially lower reagent and labor costs, and a faster turnaround), although its definitive clinical application depends on the adoption of standardized interpretation criteria. Advantages of the determination of this include rapid turnaround, robustness, and broad applicability (as it can be measured not only in the CSF and serum, but also in non-CSF fluids like saliva, urine, and lacrimal, though more evidence for these is needed at this moment). At the same time, disadvantages are represented by the lack of standardized interpretation, lack of specificity, and influence of serum elevations, as impaired renal function or monoclonal gammopathies can raise serum KFLC and confound CSF/serum ratios. It is worth mentioning that KFLC levels are altered secondary to steroid treatment, which might represent a drawback [3]; however, there is no evidence of a similar phenomenon occurring with the KFLC index. Another aspect worth mentioning is the synthesis of KFLC from all immunoglobulin classes, which, when produced intrathecally, mirrors not only IgG but also IgA and IgM production. Measuring the levels in the CSF therefore provides a clearer picture of humoral immune activity and extends the diagnostic utility outside MS or other Ig-G-driven conditions to other neurological pathologies involving immunoglobulin production [16].

In limited cohorts of NMOSD and MOGAD, studies have observed comparable rates of intrathecal KFLC synthesis and CSF-specific oligoclonal bands, though these frequencies are markedly lower than those documented in multiple sclerosis patients [33]. Data show that the intrathecal fraction of KFLC can differentiate myelitis caused by MS and NMOSD with a sensitivity of 88.5% and a specificity of 88.9% [3].

In addition to their well-described role in the diagnosis of multiple sclerosis, kappa free light chains and the derived K index have emerged as biomarkers with clear prognostic implications, as studies have proven their correlation with short-term disability and their role in the prediction of disease course. Higher K index values are correlated directly with the Multiple Sclerosis Severity Score (MSSS) at one year, while CSF levels of kappa free light chains were linked to disability progression according to the EDSS in 36 months [34].

It is also worth mentioning that emerging evidence indicates that gradual neurological decline occurs not only in progressive MS but also in relapsing–remitting MS, a phenomenon termed progression independent of relapse activity (PIRA) [35]. Since PIRA reflects gradual, relapse-independent disability fueled in part by intrathecal B cell activation and that OCB presence at onset is an established prediction of its occurrence, a raised KFLC index may similarly identify patients with increased PIRA risk. However, prospective studies are still needed to validate its accuracy in this specific setting [36,37].

One of the proposed mechanisms for cognitive impairment in patients with multiple sclerosis has been attributed to cortical gray matter injury. As an elevated KFLC index reflects pronounced B cell activation and broad immunoglobulin production within the CNS, researchers proposed this biomarker as being the one of choice in the assessment of cognitive decline. Thus, quantifying the KFLC index at diagnosis could identify patients at higher risk for early cognitive impairment [38].

### 2.3. Neurofilament Light and Heavy Chain

Neurofilaments (Nfs) are key components of the axonal cytoskeleton, serving as structural scaffolds. They ensure the stability and radial growth of myelinated axons and support the transmission of electrical impulses [39]. With a diameter of approximately 10 nm, Nfs are classified based on molecular weight as heavy (~200 kDa), medium (~150 kDa), and light (~68 kDa).

Because the NfL is the most prevalent and soluble subunit, it represents the biomarker of choice, measured both in the serum and CSF. Under normal circumstances, low concentrations of NfL are continuously released from axons, more likely in an age-dependent fashion, with higher levels being released at older ages. They serve as markers of axonal damage, the release increasing sharply secondary to inflammatory, neurodegenerative, traumatic, or vascular injury [40].

In multiple sclerosis, NfL levels have been intensely investigated by multiple studies. It has been concluded that it is a reliable biomarker that can be correlated with disease activity, as the increase in concentration is directly linked to worsening of the disease (MS relapses). More than that, studies have proven that both the CSF and serum levels are high for 2–3 months after a relapse and then gradually decrease to baseline levels. No significant difference was found when investigating CSF and serum levels in relapsing–remitting MS versus secondary progressive MS [25]. NfL levels also have utility in predicting treatment response, as patients treated with natalizumab exhibit a threefold reduction, suggesting that this therapy not only exerts an immunomodulatory effect but may also mitigate axonal damage. Similar effects have been observed in MS patients receiving rituximab, mitoxantrone, or fingolimod [41].

In addition to their role in confirming relapses for patients already diagnosed with MS, NfLs show promising results in differentiating CNS pathology from systemic disorders. The limit of this biomarker becomes evident in the differential diagnosis of other neurological pathologies, as they have low specificity, with high levels also being identified in Alzheimer’s disease, amyotrophic lateral sclerosis, Creutzfeldt–Jakob disease, dementia with Lewy bodies, frontotemporal dementia, and mild traumatic brain injury [40].

In multiple sclerosis “mimics”—NMOSD and MOGAD—especially in the individuals that are seronegative for the specific autoantibodies (AQP4 and MOG-Ab), according to researchers, NfL levels do not represent a useful tool in the differential diagnosis. The levels measured in blood, however, seem to have a prognostic signification in seropositive patients, as high levels of NfLs are correlated with a more malignant disease course, presumably due to the pathophysiology, as it proves not only astrocytic damage but also axonal involvement. Thus, blood NfL levels are correlated with disability extent in NMOSD and MOGAD [39].

Neurofilament heavy chain (NfH) levels have been less investigated compared to the NfLs and even though the studies outlined a significant difference between relapsing–remitting MS and secondary progressive MS, this difference does not seem to have any clinical significance [25].

### 2.4. AQP4 Antibodies

Even though the first data regarding neuromyelitis optica date back to the 19th century, it was not until 2004 that the discovery of AQP4 antibodies led to a better understanding of the pathology, thereby making the differential diagnosis of multiple sclerosis more feasible.

Aquaporin-4 is a water channel found mainly on the foot processes of astrocytes at the blood–brain barrier and antibodies targeting this structure seem to represent the basis of the pathophysiology [42]. The process triggered by antibodies attacking AQP4 consists of astrocyte damage, leading to the secretion of proteins such as GFAP and S100B and inflammatory factors (TNF-alfa, interleukins) [22]. Since the disability in neuromyelitis optica is directly correlated with the attacks, patients who are seropositive for the aforementioned autoantibodies seem to have a higher risk of relapses [43].

Nonetheless, the titer cannot be reliably linked to disease activity or severity, nor with therapy response. Studies have shown that patients with low- or medium-titer antibodies follow a similar disease course to patients with high-titer antibodies. These findings suggest that it is not merely the quantity of AQP4-IgG that is important, but also the molecular diversity within its components, which may vary among patients and at different stages of the disease. Identifying additional stratifying biomarkers is therefore essential, as they could improve prognostication and inform the development of effective antibody-based therapies for NMOSD [44].

### 2.5. Glial Fibrillary Acid Protein (GFAP)

Intermediate filaments consist of a group of related genes, which are categorized into five distinct types. GFAP is the principal type III intermediate filament in astrocytes, whose main function is preserving their mechanical integrity and facilitating the thickening and elongation of astrocytic processes during astrogliosis, which is a reactive response to CNS injury. GFAP expression increases with age (the expression is higher in older patients) and there are ten main isoforms, which are differently located in both the central and peripheral nervous system [45].

The role of GFAP in demyelinating spectrum disorders is outlined by the increase in the CSF of patients with NMOSD, as it is considered a biomarker of astrocyte damage [46]. Moreover, studies have shown a difference between the CSF level of GFAP in patients with AQP4-positive NMOSD and the MOG-positive patients and MS patients, thus having the potential of being used in differential diagnosis. Additionally, as the serum levels have proven to be significantly increased in the relapse compared to the remission stage, it can be concluded that it can be a potent biomarker for the correlation with the extended disability status score (EDSS) [22].

In multiple sclerosis, GFAP levels in the CSF serve as a potential biomarker for disease progression, as they reflect the neurodegenerative process of MS. Higher levels have been identified in MS patients compared to controls and in secondary progressive MS compared to the relapsing–remitting phenotype [25,47]. However, the transition from relapsing–remitting MS to secondary progressive MS is complex and GFAP levels alone are not yet established as a definitive predictive marker for this conversion.

### 2.6. Calcium Binding Protein S100B

Discovered in the 1960s, S100B is a small calcium- and zinc-binding protein [48] primarily expressed by astrocytes and, in a smaller proportion, by oligodendrocytes and other neuronal subtypes. At physiologic concentrations, it promotes astrocyte proliferation by regulating calcium ion flow (studies have shown that it acts in an autocrine, paracrine, or endocrine manner [49]); however, at pathological concentrations—achieved secondary to astrocyte damage—it exerts neurotoxic effects through the release of inflammatory mediators, stimulation of microglia to produce nitric oxide (NO), and interaction with the receptor for advanced glycation end products (RAGE) on neurons [22,50].

Even though elevated levels are found in multiple CNS conditions (Alzheimer’s disease, Parkinson’s disease, amyotrophic lateral sclerosis (ALS), traumatic neural injury, and epilepsy [51]), its role as a biomarker in demyelinating disorders has been emphasized as secondary to the discovery of high levels in the CSF and serum of patients with multiple sclerosis and NMOSD. In multiple sclerosis, elevated S100B levels are detected in the CSF during the acute phase and evidence shows that levels decrease following immunosuppressive therapy: for example, in patients treated with natalizumab [50]. In NMOSD patients, S100B levels are elevated in both the CSF and serum, especially during relapses, these increases being linked to the severity and the extent of spinal cord lesions [22].

### 2.7. Chitinase-3-like Protein 1

Chitinase-3-like protein 1 (CH3LP1) is a secreted glycoprotein implicated in regulating inflammation, macrophage polarization, apoptosis, and carcinogenesis [52]. Astrocytic chitinase-3-like protein 1 (CH3LP1) is thought to be involved in multiple neurobiological processes, among which we mention astrocyte migration, oligodendrocyte proliferation, and neural progenitor differentiation. As data show that it is secreted by activated astrocytes in the CNS, it might represent a therapeutic approach in pathologies like Alzheimer’s disease, Parkinson’s disease, stroke, ALS, or multiple sclerosis [53].

Regarding its use as a biomarker in multiple sclerosis, the current evidence supports its potential as both a diagnostic and a prognostic marker [54]. High levels identified in the CSF have been linked to cognitive impairment, a higher number of radiologically active lesions, and disability progression. Increased levels have also been identified in the serum of patients with relapsing–remitting MS, secondary progressive MS, and primary progressive MS. Moreover, despite serum CH3LP1 levels tending to rise with disease progression, findings are inconsistent regarding its role as a therapeutic target in MS. Overall, CSF CH3LP1 holds significant potential for enhancing MS diagnosis, prognosis, and treatment monitoring, though further research is necessary to fully validate its clinical utility [53].

It has also been postulated that CH3LP1 levels can be correlated with NfL levels to differentiate between MS subtypes and predict clinical progression. As NfL levels are correlated with brain gray matter volume and CH3LP1 levels are linked to spinal cord volume, a combined assessment of the two might improve the differentiation between the phenotypes: for instance, high NfL levels and low CH3LP1 levels are more frequently observed in RRMS compared to SPMS and PPMS, while simultaneous elevations are characteristic for clinical progression in RRMS [53].

### 2.8. CXCL13

CXCL13 (C-X-C motif ligand 13), also known as B cell-attracting chemokine-1 (BCA-1) or B lymphocyte chemoattractant (BLC), was first described in the late 1990s. Its production is ensured by multiple cell types, such as follicular dendritic cells and marginal reticular cells, macrophages, and monocytes. In the CNS, it appears to be the most important molecule that interferes with the recruitment of B cells to the CSF; thus, the level of intrathecally produced CXCL13 (ICXCL13) is actively being studied as a biomarker for predicting MS activity and response to therapy [55,56].

CXCL13 has the potential to be integrated into routine clinical practice by aiding clinicians in treatment decision making for multiple sclerosis patients. This includes identifying individuals most likely to respond to therapy, assessing therapeutic outcomes, and pinpointing patients with CIS or MS who are at a higher risk for inflammatory attacks and may benefit from immunomodulatory treatments. A study by Alvarez et al. [57] demonstrated that ICXCL13 is critical for identifying optimal responders to rituximab. Furthermore, reductions in ICXCL13 levels following rituximab treatment were associated with decreased concentrations of tissue damage biomarkers, such as cerebrospinal fluid myelin basic protein and neurofilament light chain (NfL), suggesting its utility as an indicator of treatment response [58].

### 2.9. N-Acetyl Aspartate (NAA)

N-acetyl aspartate (NAA) is a molecule found predominantly in neurons and their processes, as it is secreted by neuronal mitochondria and catabolized by oligodendrocytes. Its quantification is possible using proton magnetic resonance spectroscopy (1H-MRS). In multiple sclerosis, as the brain’s metabolic profile is significantly altered, NAA appears to be a prominent peak in the 1H-MRS spectrum, as it is typically regarded as an indicator of neuronal and axonal integrity. Therefore, reductions in regional NAA levels observed in both normal-appearing tissue and white matter lesions may represent a sensitive biomarker for neuronal damage and regional demyelination [59].

It may also represent a potential biomarker for differentiation between MS and NMO, as both serum and CSF levels differ markedly between the two. Notably, high serum NAA values were observed in all MS patients [60], whereas none of the NMOSD patients exhibited such elevations, regardless of NMO-IgG status, disease activity, or immunosuppressive treatment. Despite limitations such as cohort heterogeneity, limited CSF sampling, and the absence of healthy control CSF data, the parallel trends in serum and CSF NAA suggest that serum testing alone might suffice for differentiating MS from NMO. Additionally, a positive correlation between NAA levels and disability scores in relapsing–remitting MS indicates potential functional and prognostic relevance. However, the lack of difference in NAA levels between active and inactive disease states and the dynamic nature of NAA underscore the need for further longitudinal studies to clarify its role as a biomarker in neuroinflammatory disorders [61]. Current research focuses on identifying if NAA could serve as a biomarker used for predicting conversion from radiologically isolated syndrome to multiple sclerosis, as in one study based on H-MRSI at 1.5 Tesla [62,63] it was pointed out that the NAA/Creatine ratio levels are lower in RIS than in controls. Since this could not be correlated with lesion load, brain atrophy, or other prognostic markers, further exploration is required [64].

### 2.10. Galectin-9

Another biomarker extensively studied in multiple sclerosis, precisely for the differentiation between relapsing–remitting and secondary progressive phenotypes, is represented by galectin-9. As a subtype of the family of galectins, its main role is in immunomodulation, with recent evidence of its involvement in autoimmune disorders, chronic pathologies, and even cancer [65].

Regarding the involvement in CNS demyelinating disorders, evidence highlights increased levels of galectin-9 in the cerebrospinal fluid of MS patients compared to controls and in patients with secondary progressive MS compared to patients with relapsing–remitting forms. Moreover, it appears that there is a direct correlation with the number of lesions. Thus, based on the available data, we conclude that this biomarker is useful in differentiating between these phenotypes [25,65].

### 2.11. Osteopontin

Osteopontin is another biomarker worth mentioning. It was proved to play a crucial role in the pathology of multiple sclerosis and neuromyelitis optica and was firstly described as a bone matrix protein that is secreted by several cell types, including T cells, B cells, macrophages, dendritic cells, and natural killer cells. It exerts a proinflammatory action and plays, as well, a role in secondary neurodegeneration, hence being associated with the active lesions in multiple sclerosis and predicting a worse disease course. Studies prove its involvement in acute relapses, with high levels of osteopontin having been identified in the CSF of patients compared to healthy controls. Evidence also suggests an inverse relationship between CSF osteopontin levels and the time interval since the last relapse [66,67].

Its importance is underlined, as well, by the fact that the levels decrease after disease-modifying therapies [68]. Although it does not seem to be a biomarker to use in the differentiation between MS phenotypes, as studies show increased levels both in the relapsing–remitting and secondary progressive forms, the possibility of distinguishing between remissions and relapses is worth noting [25,67].

### 2.12. Chemokines

Another potential direction in the biomarker research is represented by chemokines, which, by definition, represent “a broad group of small signaling proteins”, which are broadly categorized into homeostatic and inflammatory chemokines. In multiple sclerosis, the crossing of the blood–brain barrier is mediated through immune cells, leading to demyelinating lesions and neuronal damage [69].

The highest attention was accorded to CCL11, CXCL10, CCL2, CCL3, CCL4, and CCL5, with promising results. In patients with the relapsing–remitting form, cerebrospinal fluid analysis showed elevated levels of CCL11, CXCL10, CCL4, and CCL5, but lower levels of CCL2. Researchers found no evidence of different levels of CCL3. Based on these, the conclusion was that CCL11 and CXCL10 can be positively correlated with disease progression and, at the same time, that there is also a negative correlation between CCL11 and active lesions on the MRI, and that CCL5 is in direct relation to the number of lesions in the cervical spine, while none of the above could be linked to the lesions located in the brain or thoracic spine [70].

Therefore, chemokines represent promising biomarkers in both diagnostic and prognostic directions [69].

### 2.13. Complement System

NMOSD is particularly difficult to distinguish from multiple sclerosis in the early phases of the disease. Researchers found evidence that complement activation might be a useful tool in guiding this distinction [71,72], since complement activation plays a key role in NMOSD, particularly in AQP4-IgG-positive cases. AQP4 antibody binding initiates complement-dependent cytotoxicity (CDC), causing astrocyte, neuron, and myelin sheath damage [22]. Attention has been brought to C4d and the serum C5-C9 complex, which appear to be elevated in NMOSD patients compared to healthy controls and MS patients. Other studies also showed that the C5-C9 complex is increased in both the serum and CSF, consequently highlighting the potential use as a biomarker. Moreover, therapeutic implications have been outlined in AQP4-IgG NMOSD regarding the use of eculizumab in relapse prevention. Complement fractions C3 and C4 show promising evidence of their use as biomarkers in distinguishing between MOGAD and NMOSD, since their levels are lower or comparable (C3) and, respectively, lower (C4) compared to patients with MOGAD [73].

### 2.14. Cytokines

IL-6 is a multifunctional cytokine released in response to tissue injury or infection. It exerts a physiological role in promoting B cell differentiation into plasma cells and supporting T cell survival [74,75]. Through the involvement in the formation of Th17 cells (while inhibiting regulatory T cell formation), it also exerts a pro-inflammatory response. In demyelinating disorders of the CNS, high levels of this biomarker are identified in both the CSF and serum of patients with NMOSD [10]. Therefore, recent studies centered on the implications of cytokines in the pathogenesis of demyelinating spectrum disorders, more specifically on targeting IL-6 (which is linked to the risk of relapses and higher EDSSs in patients with NMOSD), found that tocilizumab and satralizumab demonstrated clinical benefits [75,76,77]. Satralizumab seemed to be more effective in AQP4 IgG NMOSD compared to seronegative patients, while tocilizumab reduced the risk of recurrence in both NMOSD-seropositive patients and MOGAD patients [73].

### 2.15. HERV-W Peptides

Another future perspective is assured by human endogenous retrovirus (HERV), a biomarker that has been studied especially in MS patients. Researchers proved its potential in differentiating between the demyelinating disorders of the CNS since evidence shows that 78% of patients with MS are positive, compared to only 8% of patients with NMOSD (either seropositive or seronegative for AQP4). Moreover, when comparing patients with MOGAD to the ones with NMOSD, numbers also show a significant difference: 91% positivity in patients with MOGAD and 32% positivity in patients with NMOSD [73].

### 2.16. Tau

Tau, a neuronal microtubule-associated phosphoprotein, has been studied in demyelinating disorders of the CNS, with increased CSF levels being identified in multiple sclerosis patients [78]. This biomarker reliably marks axonal damage and the onset of disability, and its levels fluctuate according to both inflammatory flare-ups and neurodegenerative decline. Tau pathology and innate immunity engage in a bidirectional interaction: tau aggregates trigger microglial inflammasomes (such as NLRP3 and cGAS-STING), leading to pro-inflammatory cytokine production, while the resulting inflammation drives tau’s post-translational modifications and further aggregation [79].

Across the data available up to this date, tau in MS serves as a prognostic marker. While total tau is modestly elevated in MS compared with controls, its main role lies in reflecting ongoing axonal injury and predicting early disability accumulation [80]. Likewise, CSF tau measured at diagnosis correlates with short-term disability progression (Multiple Sclerosis Severity Score—MSSS), underscoring its role as a prognostic indicator of neurodegeneration [78].

Although identified in the early 2000s, tau has yet to be included into standard clinical protocols for MS [80].

### 2.17. Vitamin D

Due to its role in immune and central nervous system cell homeostasis, vitamin D has been extensively studied in demyelinating disorders, especially in multiple sclerosis, as a predictive biomarker.

Vitamin D may exert neuroprotective effects in MS by promoting the activation of neurotrophic factors and attenuating Th-cell-driven immune responses, with low levels being linked to diagnosis or an increased number of lesions on MRI [81]. Large observational cohort studies showed that higher 25-hydroxy-vitamin D levels predict a quieter disease course (with a lower relapse risk and a lower risk of new MRI lesions), while two of the first powered phase 3 randomized control trials have reported modest benefits. The arguments behind the mismatch include the confounding and reverse causality, as inflammation itself may lower vitamin D levels, and more disabled or sun-avoiding patients appear vitamin D deficient. Furthermore, heterogeneity—in baseline disease activity, concomitant DMTs, and vitamin D dosing regimens and duration—adds additional complexity [82].

Even though there is still need for extensive studies, data available up to this moment show that maintaining adequate vitamin D levels is an essential add-on therapy of MS care [82,83].

An exhaustive, methodical summary of the biomarkers described above is presented in the table below- Table 2 [84].

## 3. Discussion

Demyelinating disorders of the CNS remain up to this day a subject of interest in the research field due to the ongoing challenges in accurate diagnosis and therapeutic approach. Within the biomarker domain, it is essential to consider their applicability in the clinical versus research contexts, their intrinsic limitations, and their accessibility. We have briefly reviewed these elements in the following table- Table 3 [27,33,40,43,47].

**Figure 2:**
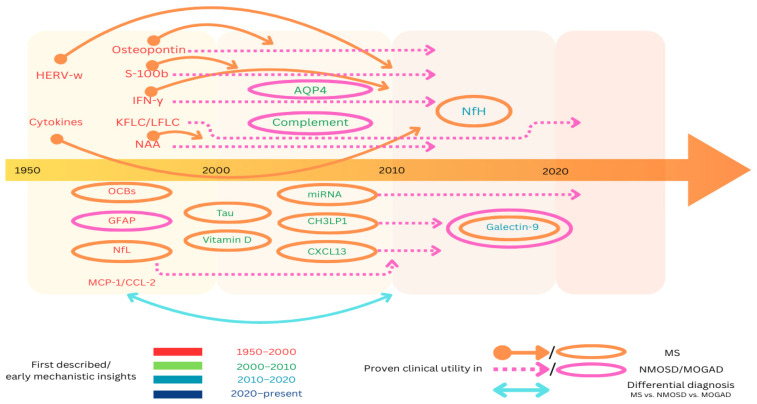
Evolution timeline of biomarkers: a schematic depiction illustrating the temporal relationship between the initial discovery of the biomarkers and their subsequent incorporation into the diagnostic protocols MS, NMOSD, and, respectively, MOGAD. The circles denote the biomarkers that were integrated into clinical practice concurrently with their discovery, while the arrows delineate the interval between their initial description and the commencement of their diagnostic application.

The temporal framework detailed below extensively describes the impact that novel technologies had on the discovery of biomarkers ever since the earliest data became available in the literature, with insights into the present and future- Figure 2.

*1950–2000s:* The first published data in the biomarker research field correspond to this period. Oligoclonal bands were first described in the early 1960s as indicators of intrathecal IgG synthesis, followed by the recognition of glial fibrillary acid protein as a structural astrocytic protein. Later on, between the 1980s and the 1990s, researchers described N-acetyl aspartate and found that its reduced levels were linked to lesions in multiple sclerosis [85]. Early studies showed that IFN-γ levels in the CSF and blood increased during relapses [86]. Controversial therapeutic trials later brought to light its role as a pro-inflammatory molecule [87]. Additionally, elevated levels of kappa and lambda free light chains were correlated with intrathecal IgG synthesis. Another biomarker first described during this decade was S100B protein: a marker of astrocyte activation and brain injury with increased levels being observed during MS relapses [49,51]. The last decade of the 20th century was marked by the discovery of MCP-1/CCL 2, with a role in neuroinflammation and blood–brain barrier disruption [88], the discovery of osteopontin, with early studies of elevated CSF/serum osteopontin levels in multiple sclerosis [89], and the description of neurofilament light chains, which only gained interest later on, with the early studies being limited by assay sensitivity.

*2000–2010s:* During the early 2000s, advances in technology led not only to the addition of several biomarkers in the diagnosis of demyelinating disorders but also to deepening the understanding of the previously known ones. A breakthrough was represented by the discovery of AQP4, which revolutionized the diagnosis of NMOSD and still represents up to this day the diagnostic gold standard. Consequently, AQP4 antibodies were proven to activate the complement system, leading to research in this direction [90]. Moreover, in the early 2000s CXCL13 was first described, with initial studies linking this molecule to intrathecal B cell activity and oligoclonal bands in MS [91,92]. Another biomarker linked to neuroinflammation and identified in patients with multiple sclerosis is chitinase-3-like protein 1, with elevated levels detected in both the CSF and serum of MS patients. Regarding the previously described biomarkers, studies were consistent with the validation of NAA predictive value for long-term disability in MS [93], and the association of MCP-1/CCL2 with active inflammation (based on MRI gadolinium-enhancing lesions and blood–brain barrier disruption). In NMOSD, GFAP-level elevations in the CSF and serum were noticed during attacks, and correlated with optic neuritis and myelitis severity [94,95]. Progress has been made as well regarding the knowledge of IFN-γ with the description of regulatory effects in progressive MS in addition to the already-described pro-inflammatory actions. Studies also confirmed serum/CSF S100B protein increasing during relapses in multiple sclerosis and, as an important addition in MS diagnosis, we note the establishment of the kappa free light chain index as a quantitative alternative to OCBs [96,97].

*2010–2020s:* This decade was particularly important due to significant data validation regarding prognostic values and the emergence of important therapeutic options. The biomarker list was completed with the addition of galectin-9 and validation of neurofilament heavy chains (first studied during the 1990s with the recognition of their role as a marker of neuronal injury). Galectin-9 was proven to play a crucial role in immune regulation (via T cell immunoglobulin and mucin-domain protein 3), with later evidence of elevated levels in the CSF of patients with multiple sclerosis and NMOSD [98,99]. The aforementioned improvement in therapeutic options was assured by the approval of eculizumab for NMOSD in 2019 [100] and by evidence linking MCP-1/CCL2 levels to therapeutic responses to IFN-β [101,102]. Significant progress has also been made predicting the conversion from clinically isolated syndrome to multiple sclerosis based on measuring kappa and lambda FLCs [103,104], with high levels not only predicting the conversion but also being correlated with MRI lesion burden and response to B cell therapies. Similar results were obtained for CXCL13. Conversion from relapsing–remitting multiple sclerosis to secondary progressive multiple sclerosis was associated with high levels of chitinase-3-like protein 1 in the CSF, which were also proved to be linked to faster disability progression [105]. Studies focusing on GFAP showed lower levels of this biomarker in MOGAD [106,107], thus giving crucial insight into the differential diagnosis of multiple sclerosis and NMOSD.

*The 2020s:* The current focus of researchers is mainly directed at therapeutic approaches. Satralizumab and inebilizumab have been recently approved in the treatment of NMOSD [108,109,110], while temelimab, an IgG4 monoclonal antibody targeting the endogenous retroviral envelope protein HERV-W-Env, is currently under development as a disease-modifying therapy for multiple sclerosis [111,112,113]. Another lead for research is focused on AQP4/AQP4 immunocomplexes, as in one study it is stated that, in the presence of activated complement, these promote the differentiation of NKT cells into the follicular helper phenotype. Consequently, this leads to a pronounced Th17 cytokine release, which could represent a potential target for therapeutic intervention [114].

Ongoing research is focused on integrating chitinase-3-like protein 1, galectin-9, MCP-1/CCL2, and GFAP as alternative biomarkers to conventional ones. Osteopontin is also under study since it was proven to play a part in myelination, with the researcher’s current focus being on defining the exact role of its isoforms (secreted and intracellular) and determining if they could represent a therapeutic target [115,116,117,118].

Another future perspective comes from the “-omics” approach, with researchers investigating whether composite algorithms (integrating proteomics, transcriptomics, and metabolomics) could serve as tools in diagnosis and tailoring therapeutic strategies. Up to this day, there is promising evidence regarding the stratification of healthy individuals from multiple sclerosis patients, of patients with RRMS who are at risk for disease progression, and even of multiple sclerosis patients from NMOSD patients [119].

Multi-omic assessment has been used in the examination of patients undergoing treatment with an anti-CD20 monoclonal antibody, ocrelizumab, concluding that ocrelizumab’s clinical efficacy is mediated through a complex network of biological pathways that extend beyond the immediate downstream consequences of B cell depletion [120].

Metabolic analysis is currently used since there is growing evidence of lipid profiles and fatty acids differentiating directly between the recurrent–remissive and secondary progressive phenotypes [119]. Despite future research still being needed, developing algorithms including “-omics” layers and clinical and imagining data might enable precision diagnosis and personalized therapy selection.

Although miRNAs are encompassed by the domain of transcriptomics, these biomarkers deserve focused attention since the latest research proves their role in modulating the immune and central nervous system processes [121]. They are a family of short, non-coding RNA molecules, typically 18–25 nucleotides long [122]. Owing to the remarkable stability of the biological fluids, they confer numerous advantages as biomarkers for multiple sclerosis [123]. Although the specific miRNA species implicated in multiple sclerosis pathogenesis have not yet been fully delineated—and additional data are required—their involvement in disease physiopathology has been unequivocally demonstrated through activation of T lymphocyte and macrophage subsets, disruption of blood–brain barrier permeability, and immunologically mediated myelin sheath destruction [121]. Moreover, several studies have shown a role for these miRNAs in remyelination [123], and current research is aimed at determining whether miRNAs might serve as biomarkers for distinguishing multiple sclerosis phenotypes and, ultimately, whether targeted therapies can be developed. Although evidence indicates that miRNAs can be employed to discriminate between NMOSD and MS [122], the same cannot yet be asserted for MOGAD, which therefore represents a promising area for future investigation.

In the context of all three conditions—multiple sclerosis, neuromyelitis optica, and myelin oligodendrocyte glycoprotein antibody-associated disease—integrated biomarker platforms that synthesize fluid-based, imaging, and immunological indicators constitute the frontier of biomarker research.

## 4. Conclusions

Historical and clinical data on multiple sclerosis and neuromyelitis optica spectrum disorder extend back more than a century, whereas MOG antibody-associated disease has only recently been incorporated into the spectrum of central nervous system demyelinating disorders. Owing to the substantial clinical burden they impose on patients, these conditions have become a primary focus of global research efforts, encompassing both the development of therapeutic interventions and the optimization of diagnostic algorithms.

Our narrative review highlights key milestones in biomarker evolution and addresses major clinical concerns: identifying which biomarkers best predict the conversion from CIS to MS, which are the most accurate for differentiating MS, NMOSD, and MOGAD, which correlate with disease severity, and which may serve as novel therapeutic targets.

Based on the available information, we summarize that during the past decades several transformative milestones in fluid biomarker research for CNS demyelinating disorders have occurred. These include the discovery and clinical adoption of AQP4-IgG, recognition of MOG-IgG as a distinct disease marker, incorporation of KFLC into diagnostic criteria, emergence of astrocytic markers (GFAP, CH3LP1, S100B), targeted cytokine therapies informed by IL-6 profiling, and the first “omics”-based and multi-modal biomarker panels.

However, biomarker research in CNS demyelinating disorders still faces several key gaps: double-seronegative patients (a subset of NMOSD and MOGAD cases test negative for both AQP4-IgG and MOG-IgG, making diagnosis and early treatment decisions challenging), a lack of standardized cut-offs and interpretation (for biomarkers like KFLC and the K index, CXCL13), low specificity for many markers, limited clinical integration of promising markers, technical and logistical barriers, and integration of multi-omic data.

Addressing these gaps will require multicenter longitudinal cohort studies that couple serial sampling with standardized imaging and clinical and treatment data. Furthermore, the development of integrated biomarker panels represents a strategic priority for future research; collectively, these initiatives may have the potential to catalyze substantial advances in the diagnosis and management of CNS demyelinating disorders.

## Figures and Tables

**Table 1:** Main features of demyelinating disorders of the CNS [4,10,11,12,13,14,15].

	Multiple Sclerosis (MS)	Neuromyelitis Optica Spectrum Disorder (NMOSD)	Myelin Oligodendrocyte Glycoprotein Antibody Disease (MOGAD)
**Key Pathophysiology**	Primarily T cell-mediated autoimmune demyelination in the CNS; B cells also play a role.	Autoimmune astrocytopathy primarily mediated by aquaporin-4 (AQP4) IgG, leading to secondary demyelination.	Autoimmune demyelination mediated by IgG targeting MOG on oligodendrocytes.
**Typical Age of Onset**	20–40 years (can vary); often a disease of young adults.	40–60 years, but can vary widely; some patients present later in life.	20–30 years; children are particularly predisposed.
**Epidemiology**	The female–male ratio is about 2–3:1 (varies by geographic region).	Marked female predominance, up to 9:1.	Nearly equal distribution.
**Clinical Presentation**	Focal motor or sensory deficits; ataxia; unilateral optic neuritis; bladder and bowel dysfunction; sexual dysfunction; cognitive dysfunction.	Severe attacks of optic neuritis, transverse myelitis (frequently longitudinally extensive), or area postrema syndrome.	-Acute disseminated encephalomyelitis (ADEM) -> focal neurological deficits, transverse myelitis, and altered mental status; -Unilateral or bilateral optic neuritis; -Transverse myelitis.
**MRI Findings**	**Brain**: -Periventricular, juxtacortical, and infratentorial lesions with a typical ovoid aspect and ring enhancement. **Spinal cord**: -Usually, peripheral cord lesions are limited to short segments.	**Brain**: -Periventricular white matter lesions, lesions of dorsal medulla, or periependymal surfaces of the third and fourth ventricles; -Long optic nerve lesions are frequently encountered. **Spinal cord**: -Longitudinally extensive lesions (≥3 vertebral segments).	**Brain**: -Large, poorly delimited lesions; -Unilateral or bilateral thalamic or basal ganglia involvement; -Longitudinally extensive optic neuritis. **Spinal cord**: -Lesions can be extensive; “H-sign” or “ventral sagittal line” signs may come across.
**Serum Biomarker**	**OCBs** in the CSF are supportive, but not definitive.	**AQP4-IgG (NMO-IgG)**-positive in ~70–80% of NMOSD patients.	**MOG-IgG** is positive in a significant proportion of patients.
**Clinical Course**	Relapsing–remitting (RR)/secondary progressive (SP)/primary progressive (PP)/progressive relapsing (PR).	Relapsing–remitting, rarely monophasic.	Relapsing–remitting or monophasic.

CNS = central nervous system; MOG = myelin oligodendrocyte glycoprotein; OCB = oligoclonal bands; CSF = cerebrospinal fluid.

**Table 2:** Overview of the biomarkers described in this review [84].

Biomarker	Category	Function/Role	Diseases and Notes
OCBs	Humoral Marker	Indicative of intrathecal IgG (and sometimes IgM) synthesis; crucial for MS diagnosis and prognosis.	**MS**: Present in over 95% of patients. **NMOSD**: Low positivity (10–25%).
AQP4-IgG	Humoral Marker	Autoantibody against aquaporin-4 water channels on astrocytic endfeet; triggers complement-mediated injury.	**NMOSD**: Highly specific marker and associated with relapse risk.
MOG-IgG	Humoral Marker	Autoantibody targeting MOG; defines a distinct disease entity.	**MOGAD**: Characteristic marker; also found in AQP4-negative NMOSD cases.
HERV-W Peptides	Humoral Marker	Derived from endogenous retroviruses; potential role in disease pathogenesis and differentiation.	**MS**: High positivity compared to NMOSD and MOGAD, aiding differential diagnosis.
KFLC and KFLC Index	Humoral Marker	Measures free light chain synthesis in the CSF; quantifies intrathecal antibody production.	**MS**: Useful in diagnosis and predicting recurrence; helps differentiate from NMOSD.
Galectin-9	Humoral Marker	Immunomodulatory glycoprotein; elevated levels correlate with increased lesion load and aid in distinguishing secondary progressive (SPMS) from relapsing–remitting MS (RRMS).	**MS**: Elevated in the CSF, particularly higher in secondary progressive MS compared to relapsing–remitting MS, assisting in phenotypic differentiation.
**Complement (C4d, C5-C9, C3/C4)**	Humoral Marker	Activation cascade triggered by AQP4-IgG binding; mediates complement-dependent cytotoxicity leading to astrocyte, neuron, and myelin damage.	**NMOSD**: Elevated complement markers (e.g., C4d, C5-C9 complex) compared to MS; the therapeutic target (e.g., eculizumab); differences in C3/C4 levels help differentiate NMOSD from MOGAD.
Neurofilaments (NfL, NfH)	CNS-Related Marker	Structural proteins released from axons upon injury; indicate axonal damage and neurodegeneration.	**MS**: Elevated during relapses and correlates with disability. **NMOSD and MOGAD**: High levels indicate severe axonal involvement.
GFAP	CNS-Related Marker	Astrocytic intermediate filament; marker of astrocyte damage and reactive gliosis.	**MS**: Associated with disease progression and neurodegeneration. **NMOSD**: Elevated during attacks (optic neuritis, myelitis).
S100B	CNS-Related Marker	Calcium-binding protein; supports astrocyte proliferation at physiological levels but is neurotoxic at high concentrations.	**MS and NMOSD**: Elevated during acute phases/relapses, contributing to neuroinflammation.
CH3LP1 (Chitinase-3-Like Protein 1)	CNS-Related Marker	Secreted by activated astrocytes; correlates with lesion load, cognitive impairment, and progression.	**MS**: Serves as a diagnostic/prognostic marker; higher levels indicate clinical progression.
NAA (N-Acetyl Aspartate)	CNS-Related Marker	Neuronal metabolite reflects neuronal and axonal integrity; reduction indicates neuronal loss and demyelination.	**MS**: Reduced in lesions; differential levels help distinguish MS from NMOSD.
Tau	CNS-Related Marker	Microtubule-associated protein released upon axonal and neuronal injury; reflects neurodegeneration.	**MS**: CSF total tau is elevated, correlates with disease progression.
CXCL13 and Related Chemokines (e.g., CCL11, CXCL10)	Cell Marker	Chemokines that mediate immune cell recruitment (e.g., CXCL13 recruits B cells) and promote inflammation.	**MS**: Elevated levels correlate with disease activity, lesion burden, and treatment response.
**Osteopontin**	Cell Marker	Proinflammatory glycoprotein secreted by various immune cells; involved in active lesion formation and secondary neurodegeneration.	**MS**: Elevated during relapses. **NMOSD**: Increased but less specific for phenotype differentiation.
IL-6	Cytokine	Pro-inflammatory cytokine; drives immune activation and modulates relapse risk and severity.	**NMOSD**: High IL-6 levels are linked to relapse risk and higher disability scores; also implicated in MOGAD.
Vitamin D	Nutritional/immunological biomarker	Lipid-soluble vitamin, exerts hormone-like effects.	**MS**: Low vitamin D levels in early RRMS predict a greater risk of exacerbations and MRI activity.

OCBs = oligoclonal bands; MS = multiple sclerosis; NMOSD = neuromyelitis optica spectrum disorder; AQP4 = aquaporin-4; MOG = myelin oligodendrocyte glycoprotein; HERV = human endogenous retrovirus; KFLC = kappa free light chain; CSF = cerebrospinal fluid; NfL = neurofilament light chain; NfH = neurofilament heavy chain; MOGAD = myelin oligodendrocyte glycoprotein antibody disease; GFAP = glial fibrillary acidic protein; S100B = calcium binding protein; CH3LP1 = chitinase-3-like protein 1; NAA = N-acetyl aspartate; CXCL13 = (C-X-C) motif ligand 13; IL-6 = interleukin-6; RRMS = recurrent–remissive multiple sclerosis.

**Table 3:** Additional aspects of the biomarkers reviewed [27,33,40,43,47].

Biomarker	Application	Limitations	Accessibility
OCBs	Incorporated into McDonald criteria as a diagnostic substitute for dissemination in time; routine CSF test in MS.	Low specificity (present in other neuroinflammatory diseases); transient or low-level positivity in NMOSD; requires lumbar puncture.	Widely available.
KFLC and KFLC Index	Included in 2024 McDonald criteria as alternative evidence of intrathecal Ig synthesis; emerging prognostic marker.	Lack of universally accepted cut-offs; serum elevations (e.g., renal impairment) can confound; steroid treatment may affect levels; limited specificity outside MS.	Available in many immunology labs; faster and less labor-intensive than OCBs.
Neurofilaments (NfL, NfH)	Serum NfL increasingly used to monitor disease activity and treatment response.	Low specificity (elevated in various neurodegenerative and acute CNS injuries); neurofilament heavy chains (NfHs) are less clinically validated.	Requires ultra-sensitive assays; available in reference laboratories.
AQP4 IgG	Gold-standard diagnostic biomarker for NMOSD.	Titer does not reliably reflect disease activity or severity; double-seronegative cases remain challenging.	Widely available.
MOG-IgG	Diagnostic marker for MOGAD; distinguishes MOGAD from AQP4-NMOSD and MS.	Variable assay sensitivity and specificity across platforms; seronegative cases possible.	Available in specialized/reference labs; less widespread than AQP4-IgG.
GFAP	Research marker of astrocytic damage; potential differentiation between NMOSD, MOGAD, and MS phenotypes.	Age-dependent expression; multiple isoforms; overlap between diseases; not used clinically.	Measured in research settings; not routine.
**S100B**	Research marker of acute astrocyte activation in relapses; correlates with lesion severity.	Low disease specificity (elevated in many CNS pathologies); biphasic effects (neurotrophic vs. toxic); not used clinically.	Measured in research settings.
**CH3LP1**	Research diagnostic/prognostic marker in MS; correlates with lesion load, cognitive decline, and progression.	Inconsistent therapeutic-monitoring data; overlap with other neuroinflammatory conditions; not used clinically.	Measured in research settings.
CXCL13 and Related Chemokines	Research tool to predict B cell–driven activity and treatment response in MS.	Short half-life; influenced by concurrent infections; lack of normative ranges; not in guidelines.	Measured in reference labs.
NAA (N-Acetyl Aspartate)	Research imaging biomarker via spectroscopy for neuronal integrity; potential in differential diagnosis (MS vs. NMOSD).	Requires specialized MR spectroscopy hardware and expertise; influenced by scanner field strength; not routine.	Available at centers with spectroscopy capability.
Galectin-9	Research marker to distinguish SPMS from RRMS; correlates with lesion burden.	Limited longitudinal data; overlap with other autoimmune diseases; not validated clinically.	Measured in research settings.
**Osteopontin**	Research marker of acute relapses and lesion activity in MS and NMOSD; potential treatment-response indicator.	Elevated in both RRMS and SPMS (limited phenotypic specificity); influenced by systemic inflammation; not clinical.	Measured in research settings.
**Complement Components (C4d, C5-C9)**	Research differentiation of NMOSD vs. MS/MOGAD; therapeutic target.	Complement activation is common to many conditions; requires CSF/serum; dynamic and labile.	Measured by specialized immunoassays in a few labs.
IL-6	Research marker of relapse risk in NMOSD; guides anti-IL-6 therapies.	Levels vary with systemic inflammation; pre-analytical instability.	Available in clinical immunology labs.
**HERV-W**	Research marker to differentiate MS from NMOSD/MOGAD; potential therapeutic target.	Novel; assays not standardized; unclear pathophysiological specificity.	Measured in research settings.
Tau	Research prognostic marker of early disability accumulation in MS; reflects axonal injury.	Low specificity (elevated in various neurodegenerative diseases); dynamic relationship with inflammation; not in MS protocols.	Not routine for MS; serum assays experimental.
Vitamin D	Routinely measured as an add-on to monitor supplementation in MS.	Not diagnostic.	Widely available in clinical labs; serum 25-OH vitamin D assay routine.

OCBs = oligoclonal bands; MS = multiple sclerosis; NMOSD = neuromyelitis optica spectrum disorder; AQP4 = aquaporin-4; MOG = myelin oligodendrocyte glycoprotein; HERV = human endogenous retrovirus; KFLC = kappa free light chain; CSF = cerebrospinal fluid; NfL = neurofilament light chain; NfH = neurofilament heavy chain; MOGAD = myelin oligodendrocyte glycoprotein antibody disease; GFAP = glial fibrillary acidic protein; S100B = calcium binding protein; CH3LP1 = chitinase-3-like protein 1; NAA = N-acetyl aspartate; CXCL13 = (C-X-C) motif ligand 13; IL-6 = interleukin-6; RRMS = recurrent–remissive multiple sclerosis; SPMS = secondary progressive multiple sclerosis; MRI = magnetic resonance imaging.

## Data Availability

The raw data supporting the conclusions of this article will be made available by the authors without undue reservation.
